# Preferential Impairment of Auditory Working Memory in Long COVID: An Observational Study of Undergraduate Medical Students

**DOI:** 10.7759/cureus.51457

**Published:** 2024-01-01

**Authors:** Soumen Manna, Shaon Ghosh Dastidar, Ramkumar S, Himani Ahluwalia, Manpreet Kaur

**Affiliations:** 1 Physiology, Vardhman Mahavir Medical College and Safdarjung Hospital, New Delhi, IND

**Keywords:** post-covid symptoms, long covid, covid 19, visuospatial working memory, attention span

## Abstract

Background

Long COVID is a multisystem condition with prolonged symptoms that develop after recovery from the COVID-19 infection, often following a mild infection. Few studies have been conducted on cognitive function among medical students after recovery from mild COVID-19*.* This study aimed to assess the attention span and working memory (WM) capacity of medical students after six months of recovery.

Methods

A cross-sectional study was performed on 17 young adult medical students who had suffered a mild COVID-19 infection at least six months prior. Eighteen age-matched healthy medical students served as the controls. Audio-visual WM tasks and attention spans were assessed using computerized software for both the cases and controls.

Results

The mean ages of the case and control were 19.67±1.6 and 20.0±1.2 years, respectively. The most common symptoms among cases were fatigue (33%), weight loss (26%), and nasal stuffiness (13%). The overall proportion of correct responses across all visual and auditory WM tasks (p=0.085) and reaction times (p=0.609) did not differ between the cases and controls. However, the overall target hit rate of the auditory WM task was significantly lower in cases than in controls (p=0.002). This difference was not observed in the visual WM task (p=0.374).

Conclusion

In the current study, the overall WM functions (visual and auditory combined) and attention span did not differ between cases and controls. However, auditory WM performance was significantly impaired in patients compared with controls, indicating selective impairment of auditory WM in patients with long COVID.

## Introduction

The coronavirus disease-19 (COVID-19) pandemic has affected more than four crores of Indians over two and a half years. In India, the first wave of COVID-19 began in March 2020 and lasted around November 2020, whereas the second wave lagged behind the Western world and lasted from March 2021 to May 2021 [1].

Long COVID is a multisystem condition of prolonged symptoms that develop following a COVID-19 infection, often following a mild infection [2]. Long COVID affects all age groups, and most cases occur in non-hospitalized patients with mild acute illnesses [3]. Central nervous system (CNS) involvement has been of particular interest because of its potential implications for cognitive function and various chronic neuropsychiatric sequelae, even after a mild COVID-19 infection [4]. Prominent long-lasting neuropsychiatric sequelae after COVID-19 infection known as “brain fog" are characterized by impaired memory, attention, processing speed, and executive functions [5].

Medical students are at a higher risk of contracting COVID-19 and may be a source of epidemic clusters in different hospital wards [6]. The prevalence of COVID-19 among medical students varies across regions. In a study at the University of Jordan, it was shown that clinical medical students (15.2%) are affected more than pre-clinical students (11.2%) [6]. The majority of COVID-19 cases are mild, and screening tests are the only way to find them [7]. Medical students who had been infected with COVID-19 reported higher levels of anxiety, depression, insomnia, and post-traumatic stress [8]. The challenges faced by students during the pandemic may contribute to their cognitive impairment, including attention and memory deficits [9].

Few studies have also reported mild impairment of short-term memory and attention in a comparatively younger population, even after four months of recovery [10-12], and a study by Omar et al. documented impaired memory and attention function among doctors and healthcare workers (HCWs) [13].

Long-term cognitive deficits are detrimental to individuals' day-to-day functioning, especially among young medical students, who require optimal functioning cognitive skills to complete educational degrees, work performance, and social interactions. However, few studies have assessed the working memory (WM) and attention span of young medical students six months after a mild COVID-19 infection.

Therefore, the objective of this study was to assess the attention, visual, and auditory WM of young medical students after recovery from mild COVID-19.

## Materials and methods

This cross-sectional, observational study was conducted at Vardhman Mahavir Medical College & Safdarjung Hospital, New Delhi, India, over five months (April 2022 to July 2022) after obtaining ethical clearance from the Institutional Human Ethical Committee (IEC/VMMC/SJIH/Project/2022-02/CC-236).

Seventeen post-COVID patients (cases) and 18 age-matched healthy subjects (controls) were included in this study. The sample size calculation was performed using the memory function of COVID-19 patients in comparison with healthy controls from a reference article by Akinc et al. [10]. The minimum sample size with a confidence interval (2-sided) of 95% and 80% power was 34 (17 in each group). The sample size was calculated using Epi Info, Centers for Disease Control and Prevention (CDC) [14].

Consecutive patients were recruited from undergraduate medical students at our medical college using a convenience sampling method. The inclusion criteria were (i) history of COVID-19 infection documented by a positive RT-PCR report at least six months prior, (ii) age between 18 and 25 years, and (iii) right-handedness. The general exclusion criteria were: (i) history of moderate or severe COVID-19 infection requiring oxygen supplementation or hospitalization during the acute course of the COVID-19 infection; and (ii) history of CNS disorders or subjects taking antipsychotics, antidepressants, and antiepileptic drugs that may interfere with the assessment. In addition, controls were recruited from age-matched undergraduate medical students with similar socioeconomic status and without documented COVID-19 infection, history of COVID-19 disease-like symptoms during the second wave, or any history of major medical or surgical disorders.

Following recruitment and obtaining written informed consent, demographic data and clinical history including current symptoms were collected using a questionnaire on ‘Google Forms' designed for all participants. The participants were then acquainted and asked to perform a computer-based dual-task n-back task (DTNBT) and a continuous performance test-identical pair (CPT-IP) using the Inquisit 6 software (Milliseconds, USA; https://www.millisecond.com/). The DTNBT is a go/no-go visual and auditory WM performance task with increasing levels of difficulty, whereas the CPT-IP measures the continuous and selective attention of the participants.

All evaluations and recordings were performed between six and eight months (201±26 days) after the diagnosis of COVID-19 by RT-PCR. Evaluations and recordings of the control group were also done at the same time.

In the DTNBT, the participants were presented with two sequences of stimuli in two modalities simultaneously: (a) visual stimulus, a random sequence of blue squares that can be presented in eight different locations on the computer screen, and (b) auditory stimulus, a random sequence of eight spoken Roman letters heard through noise-cancelling headphones fitted to the participant’s ear. The details of DTNBT were discussed in our previous article [15,16]. Briefly, participants were asked to respond according to the following criteria: for 1-back (N=1) trials, if the location of the blue square was the same as the one in the previous trial, then it's a target, and the participant had to press ‘A’ on the computer keyboard; if not, then he/she did not have to press ‘A’. Similarly, if the auditory letter was the same as in the previous trial, then it's a target, and the participant had to press ‘L’ on the computer keyboard; if not, he/she did not have to press ‘L’. Similarly, 2-back and 3-back trials were performed by the participants.

In the CPT-IP, the participant’s task was to identify identical pairs of four-digit numbers, presented as a continuous stream, one at a time, in the middle of the screen at a fairly fast presentation rate. The task was to press the 'space bar’ key on the computer keyboard any time if successive numbers were repeated (go trials). For non-repeating stimuli, including lures that looked similar to the previous stimulus (catch stimulus), the participants were instructed to wait for the next target (non-go trial). The total time required to complete the test was approximately three minutes.

The summary data file of the CPT-IP test contained the numbers of the target ‘hit rate’, target ‘miss rate’, ‘false alarm’ rate of catch stimulus, ‘correct rejection’ rate of catch stimulus, mean reaction time (RT) of hits, and mean RT of false alarms.

Statistical analysis

The data were compiled and analyzed using the statistical software GraphPad Prism 9. The data were first checked for normality of distribution using the Kolmogorov-Smirnov test. Demographic data is presented as mean and median. Thereafter, tests for the statistical significance of the quantitative variables between the two groups were carried out using an unpaired Student’s t-test for normally distributed parameters and a Wilcoxon signed rank test for non-parametric parameters. The significance level was considered as a p-value <0.05, a confidence interval of 95%, and β = 0.2, and power was considered as 80%.

## Results

The demographic data of the participants in the case and control groups is presented in Table 1. The mean (SD) ages of the cases and control were 19.67 (1.6) and 20 (1.2) years, respectively. Fatigue (33%), weight loss (26%), nasal stiffness (13%), and dizziness while standing (6.7%) were the main symptoms in post-COVID-19 subjects at the time of testing. However, none of the post-COVID subjects reported any subjective complaints of memory impairment in the form of forgetfulness (Table 1).

**Table 1:** Demographic data of the study population BMI: body mass index; WM: working memory; CPT: continuous performance test

Description statistics	Case (n=17)	Control (n=18)
Age in years (Mean±SD)	19.67±1.6	20.0±1.2
BMI in kg/m^2 ^(Mean±SD)	22.9±1.0	22.2±2.0
Sex		
Male	40%	50%
Female	60%	50%
Socioeconomic status	Medium	Medium
History of smoking	No	No
Coexisting disease	Nil	
Day of testing (WM & CPT) after COVID-19 diagnosis: (Mean±SD)	201±26	-
Duration of symptomatic COVID-19 in days (onset of symptoms till recovery): (Mean±SD)	11.73±3.90	-
Symptoms during COVID-19 infection (in percentage):		
Fever	80.0	-
Headache	66.7	-
Fatigue	86.7	-
Anosmia (loss of smell)	73.3	-
Dysgeusia (loss of taste)	46.7	-
Shortness of breath	26.7	-
Cough	53.3	-
Dizziness, vertigo	13.3	-
Palpitation	13.3	-
Anxiety	6.7	-
Diarrhoea	13.3	-
Drop in oxygen saturation (SPO_2_)	13.3	-
Treatment received during COVID-19 infection (in percentage):		
Paracetamol	80.0	-
Azithromycin	60.0	-
Ivermectin	46.7	-
Hydroxychloroquine	6.7	-
Doxycycline	6.7	-
Vitamin B & Vitamin C	6.7	-
Corticosteroids	0	-
Montelukast and levocetrizin	6.7	-
Interferon B	0	-
Lopinavir/ritonavir	0	-
Tocilizumab	0	-
Post-COVID-19 symptoms at the time of recording of data (in percentage):		
Fatigue	33.3	-
Forgetfulness	0	-
Anxiety	6.7	-
Loss of attention	13.3	-
Depression	6.7	-
Headache	6.7	-
Dizziness on standing	13.3	-
Nasal stiffness	13.3	-
Weight loss	26.7	-
Weight gain	6.7	-
Menstrual irregularities	0	-
Sleep-related problem	0	-
Vaccination status at the time of COVID-19 infection (in percentage):		
Two doses received	0	-
One dose received	46.7	-
Unvaccinated	53.3	-
Vaccination status at the time of recording of data (in percentage):		
Two doses received	80	100
One dose received	13.3	0
Unvaccinated	6.7	0

The performance of WM tasks and the attention span were compared between the case and control groups based on multiple parameters. The ‘overall proportion of correct responses’ across all visual and auditory WM tasks did not differ between the case and control groups (p =0.085) (Table 2). The visual WM tasks in terms of the target ‘hit rate’ (p=0.374) and ‘false alarm rate’ (p=0.059) didn’t differ between cases and controls. However, the overall target ‘hit rate’ (p=0.002) and ‘Z score of hit rate’ (p=0.004) were significantly lower in the cases than in the control group (Table 2).

**Table 2:** Comparison of working memory parameters between cases and control The value is in mean±SD for a parametric test and median for a non-parametric test. # Paired t-test, ^ Wilcoxon Signed Ranks Test. * When the p-value is < 0.05, it is significant; ** when it is <.01, it is highly significant.

Sl. No.	Parameters	Case (n=17)	Control (n=18)	Significance
1	Overall proportion of correct	0.73±0.07	0.77±0.03	# p=0.085
Visual Working Memory Parameters				
2	Hit rate overall	0.69±0.09	0.72±0.03	# p=0.374
3	False alarm rate overall	0.06	0.05	^ p=0.059
4	Z score of hit rate overall	0.54±0.27	0.58±0.12	# p=0.519
5	Z score of false alarm rate overall	-1.501	-1.624	^ p=0.114
6	Parametric sensitivity overall	2.06±0.51	2.23±0.19	# p=0.181
Auditory Working Memory Parameters				
7	Hit rate overall	0.74 ±0.09	0.83±0.08	# p=0.002**
8	False alarm rate overall	0.10	0.12	^ p=0.319
9	Z score of hit rate overall	0.55	1.01	^ p=0.004**
10	Z score of false alarm rate overall	-1.25	-1.16	# p=0.429
11	Parametric sensitivity overall	1.99	2.29	# p=0.034*

The parameters of the attention span tasks are listed in Table 3. However, none of the parameters of attention span testing differed between the case and control groups, including the ‘reaction time’ (Table 3). 

**Table 3:** Comparison of ‘attention span’ parameters between cases and control RT: reaction time; value is in mean±SD for parametric test and median for non-parametric test. # Paired t-test, ^ Wilcoxon Signed Ranks Test. * When p-value < 0.05, it is significant; ** when it is <.01, it is highly significant.

Sl. No.	Parameters	Case (n=17)	Control (n=18)	Significance
2	Hit rate	0.83	0.83	^ p=0.875
3	Miss rate	0.18±0.17	0.15±0.06	# p=0.393
4	False alarm	0.14	0.19	^ p=0.835
5	Correct rejection	0.85	0.80	^ p=0.733
6	Mean RT of hits	516.8±57.06	526.4±49.93	# p=0.609
7	Mean RT of false alarms	479.3	437.5	^ p=0.753

## Discussion

COVID-19 is primarily reported as a lower respiratory tract infection. However, the loss of smell and taste sensation in patients with acute COVID-19 has turned attention towards the possible role of CNS involvement [17] in COVID-19 patients. However, it has been increasingly recognized that COVID-19 has a broader impact, affecting virtually any system in the body, including the cardiovascular, renal, and central nervous systems (CNS) [18]. In addition, many studies have reported that even after clinical recovery from acute COVID-19, patients continue to experience fatigue, anxiety, depression, insomnia, and cognitive impairments, sometimes known as “brain fog” [5,19].

Studies have reported global cognitive function impairments, including attention and executive functions, in post-COVID patients [20-24]. However, there is a large heterogeneity in patient profile, disease severity, and assessment time (post-COVID days) when cognitive functions or other neuropsychiatric tests are performed. Most of these assessments were performed on patients with a history of hospitalization due to COVID-19 infection or a severe form of COVID-19 infection, and assessment of memory and attention was done within one month to three months after recovery from COVID-19 infection [21-23,25]. A systematic review of the impact of COVID-19 on cognitive function reported memory deficits; however, it could not differentiate whether mild cognitive impairment was due to COVID-related pathology or the effect of dementia [26]. Long-term assessments, particularly at six months or later after complete recovery, especially in the younger population, are very few.

In our current study, WM and attention span were assessed in 17 undergraduate medical students (mean age 19.67±1.6years) with documented mild COVID-19 infection and without any history of hospitalization or oxygen therapy during the course of COVID-19. Both WM capacity and attention span were assessed between six and eight months (mean, 201±26 days) after full recovery from mild COVID-19. Results were compared with 18-age (mean age 20.0±1.2 years) matched healthy controls without any history of COVID-19 infection.

We found that the overall WM function (combined visual and auditory) did not differ between cases and controls, as evidenced by the ‘overall proportion of correct’ responses in the DTNBT (p=0.085). When visual and auditory WM functions were analyzed separately, visual WM performance did not differ between cases and controls; however, auditory WM performance was significantly better in the control group than in the control group. We also found no difference in the attention span between cases and controls.

Similar to our findings, Al-Qahtani et al. reported cognitive impairment, depression, and anxiety among young college students in Saudi Arabia infected with mild COVID-19 [9]. Another study by Omar et al. reported lower scores for memory and attention function among healthcare workers infected with COVID-19 than among healthy controls, even after two weeks to three months of recovery from COVID-19 [13]. A few other studies have also reported a slowing of cognitive processing speed, as evidenced by low Symbol Digit Modalities Test (SDMT) scores and long-term verbal and spatial memory dysfunctions five months after recovery from COVID-19 infection [23]. A cohort study conducted in Norway reported that SARS-CoV-2 positivity at baseline was strongly associated with reporting memory problems at eight-month follow-up, with an odds ratio of 4.66 (95% CI, 3.25-6.66) compared to the untested, randomly selected group [27]. However, separate analyses of visual and auditory WM functions were not performed in these studies.

While our study provides evidence that mild COVID-19 infection selectively impairs auditory WM without affecting visual WM or attention, the underlying mechanism is unknown. The main pathophysiology of central nervous system (CNS) involvement in COVID-19 includes neuroinflammation, glial and neural dysregulation leading to neural circuit dysfunction, and persistent cognitive dysfunction [28]. Recent studies have provided evidence for the etiology of memory deficits caused by COVID-19. COVID-19 infection reduces gray matter thickness in the frontal cortex [29], which is responsible for working memory function. A recent systematic review also reported that, in addition to structural abnormalities, functional abnormalities such as hypometabolism in different brain regions, particularly in the frontal and parietal regions, lead to memory impairments among COVID-19 survivors [30]. Understanding these mechanisms is crucial for developing effective diagnostic and treatment options for individuals with long COVID and cognitive impairments. However, with the current literature, the pathophysiology of selective impairment of auditory WM after mild COVID-19 infection is yet to be understood.

Limitation

Our study included only a small number of participants. Although we used objective tasks for working memory and attention function assessment, a cognitive battery of tasks with an electroencephalogram (EEG) would be more appropriate for drawing conclusions, preferably with a large study population. Moreover, the pathophysiology of selective auditory WM dysfunction could not be predicted in the present study. 

## Conclusions

In the current study, the overall WM functions (visual and auditory combined) and attention span did not differ between cases and controls. However, selective auditory WM performance was significantly deficient in cases compared with the control group, indicating selective impairment of auditory WM without much involvement of visual WM or attention span in patients with long COVID.
